# Nondestructive, quantitative viability analysis of 3D tissue cultures using machine learning image segmentation

**DOI:** 10.1063/5.0189222

**Published:** 2024-03-28

**Authors:** Kylie J. Trettner, Jeremy Hsieh, Weikun Xiao, Jerry S. H. Lee, Andrea M. Armani

**Affiliations:** 1 Mork Family Department of Chemical Engineering and Materials Science, Viterbi School of Engineering, University of Southern California, Los Angeles, California 90089, USA; 2 Ellison Institute of Technology, Los Angeles, California 90064, USA; 3 Pasadena Polytechnic High School, Pasadena, California 91106, USA; 4 Department of Medicine, Keck School of Medicine, University of Southern California, Los Angeles, California 90033, USA

## Abstract

Ascertaining the collective viability of cells in different cell culture conditions has typically relied on averaging colorimetric indicators and is often reported out in simple binary readouts. Recent research has combined viability assessment techniques with image-based deep-learning models to automate the characterization of cellular properties. However, further development of viability measurements to assess the continuity of possible cellular states and responses to perturbation across cell culture conditions is needed. In this work, we demonstrate an image processing algorithm for quantifying features associated with cellular viability in 3D cultures without the need for assay-based indicators. We show that our algorithm performs similarly to a pair of human experts in whole-well images over a range of days and culture matrix compositions. To demonstrate potential utility, we perform a longitudinal study investigating the impact of a known therapeutic on pancreatic cancer spheroids. Using images taken with a high content imaging system, the algorithm successfully tracks viability at the individual spheroid and whole-well level. The method we propose reduces analysis time by 97% in comparison with the experts. Because the method is independent of the microscope or imaging system used, this approach lays the foundation for accelerating progress in and for improving the robustness and reproducibility of 3D culture analysis across biological and clinical research.

## I. INTRODUCTION

Cellular viability is a fundamental metric used to characterize the growth characteristics and proliferative capability of cell or tissue cultures. Viability assays are used throughout biology and preclinical toxicology to understand a wide range of behaviors, such as changes in cell growth induced by therapeutic perturbations or change in cell culture conditions. Assaying techniques range from colorimetric indicator dyes^1–4^ or fluorescence probes^5,6^ to an analysis of the metabolic activity by chemically lysing the cells to determine the amount of ATP present using luminescence.^7^ The results, typically reported from 0% to 100%, are obtained by evaluating the ratio of healthy to dead cells or the metabolic activity of healthy cells in a sample population. In some cases, cell viability assay results are supported by performing orthogonal measurements, such as sample imaging.

As the application of deep learning to image analysis has increased in the biomedical field,^8,9^ many researchers have found value in automating image analysis pipelines to extract quantitative information about the cellular systems studied.^10,11^ These computational models can segment and identify single cells and cell types,^12–14^ predict phenotypes of the cells,^15,16^ assign fluorescent markers to cell images,^17,18^ and quantify viability of the cells within a given image.^19^ The principles behind these models have enabled advances beyond single cell and apply to tissue-level analyses,^20^ medical imaging,^21,22^ and predictive diagnostics for diseases such as cancer.^23–26^ However, a weakness of both the original viability assays and the imaging techniques is that they were initially developed for use in 2D culturing conditions in flasks/dishes or multi-well plate formats.

Recent research has demonstrated that cells grown in 3D culturing systems better recapitulate the matrix interactions and cell phenotypes found in physiological tissue.^27^ The representation of these complex interactions provides an improved *in vitro* method for compound screening over 2D systems. While improvements in imaging technology have advanced the ability to capture qualitative information about 3D cell models and tissues,^28–30^ the need for longitudinal quantitative assessment of culture viability is still present. Various strategies are being pursued to address this challenge. One manufacturer has adapted their assay to create a 3D culture specific product. This product is called CellTiter-Glo 3D (CTG) and has quickly become an industry standard assay used to report viability by measuring the metabolic activity of the culture. However, the CTG development procedure requires complete cell lysis. For other methods, researchers have investigated the comparative use of previously developed assays^4^ and recommend complementary imaging for experiments utilizing 2D specific reagents to cross reference results.^31^ Although the field has begun adapting viability assays^32–34^ and deep learning techniques to the dynamic nature of 3D systems,^35,36^ quantification of viability largely requires additional experimental steps for fluorescently labeling the culture system^37,38^ or developing metabolic assays.^39,40^ This remaining challenge provides a unique opportunity to utilize deep learning to augment 3D viability assays.

In the present work, we develop and validate a Segmentation Algorithm to Assess the ViabilitY (SAAVY) of 3D cultures. We designed SAAVY to automatically identify and analyze features common to spheroid and organoid structure that experts correlate with cellular viability, such as the transparency of the spheroid and the overall morphology.^41–43^ SAAVY is designed for use with label-free optical images that are saved in the universal imaging formats (png, tiff, and jpeg), making it independent of the microscope system used to acquire the images. We trained and tested SAAVY against clear and noisy backgrounds. This approach ensured that SAAVY can withstand a degree of background noise that can arise from common biological defects, such as dead cell fragments or matrix particulate deposits. SAAVY calculates the viability of each uniquely identified spheroid in an image and an overall average viability for all 3D structures present in a well. It also reports the total spheroid count per image, spheroid radius, spheroid area, and other metrics of relevance. The total analysis time per well is approximately 0.3 s. This type of integrated analysis provides insight into a biological system's response at both the individual spheroid and entire well level. Finally, SAAVY is agnostic of microscope system or manufacturer and does not require fluorescent or colorimetric indicators, enabling longitudinal response studies to be performed.

The accuracy of SAAVY in analyzing pancreatic ductal adenocarcinoma (PDAC) spheroids in clear and noisy backgrounds was validated through a blinded comparison with a pair of spheroid analysis experts. Subsequently, a series of application-driven experiments were conducted. We first compared SAAVY analysis with an industry standard metabolic assay for 3D cultures, including spheroid expert analysis as ground truth. To challenge SAAVY's ability to detect viability changes in response to a perturbation, we then performed a label-free imaging-based longitudinal study investigating the effect of an FDA-approved therapeutic on the pancreatic cancer spheroids.

## II. RESULTS AND DISCUSSION

To segment the biological regions of interest in an image, prior image recognition approaches focused on utilizing relatively simple edge detection techniques to identify spheroid boundaries.^44,45^ However, edge detection with watershed has poor reliability when spheroids or organoids overlap or when background noise is present, limiting the utility to images with low spheroid density or complete separation of cell colonies.^40^ Other characterization workflows reduced or eliminated this overlap by altering culturing conditions (e.g., growing singe spheroids per well) or image acquisition settings (e.g., increased magnification on the sample wells to obtain one spheroid per image) on a case-by-case basis.^40,44,46^ This bespoke approach relies on image-stitching and partial data rejection of overlapping regions of interest, which can lead to unintentional bias in the final dataset.^47,48^ Our goal is to develop an algorithm that can analyze an image of an entire well with minimal data rejection while resisting the influence of background noise, which requires a different approach to segmentation.

As a proof of concept, PDAC spheroid samples are used. PDAC spheroids are of a cystic phenotype and tend to grow randomly throughout the well.^49^ This growth pattern leads to a high frequency of overlapping cell structures and makes them difficult to analyze using edge detection machine-learning approaches. Healthy cystic spheroids are distinguished by their open lumen and transparent centers with distinct, circular edges when viewed in plane. The transparency and circular morphology of healthy spheroids are in stark contrast with the opaque, blebbed spheroids that characterize dead spheroids of this type. This correlation between transparency and morphology and spheroid viability in brightfield images has been previously studied.^50,51^ For example, this characteristic indicator is seen in kidney,^52^ ectocervical,^53^ colon/intestinal,^54,55^ nasal epithelial,^56^ and liver^57^ model systems. As a part of this work, an informal survey of spheroid and organoid experts across cancer fields was performed, and they identified the same metrics. These results are included in the supplementary material. As a result, the ability to correlate opacity to spheroid viability forms the foundation of our label-free image-based quantification approach.

### A. SAAVY design

**FIG. 1.**
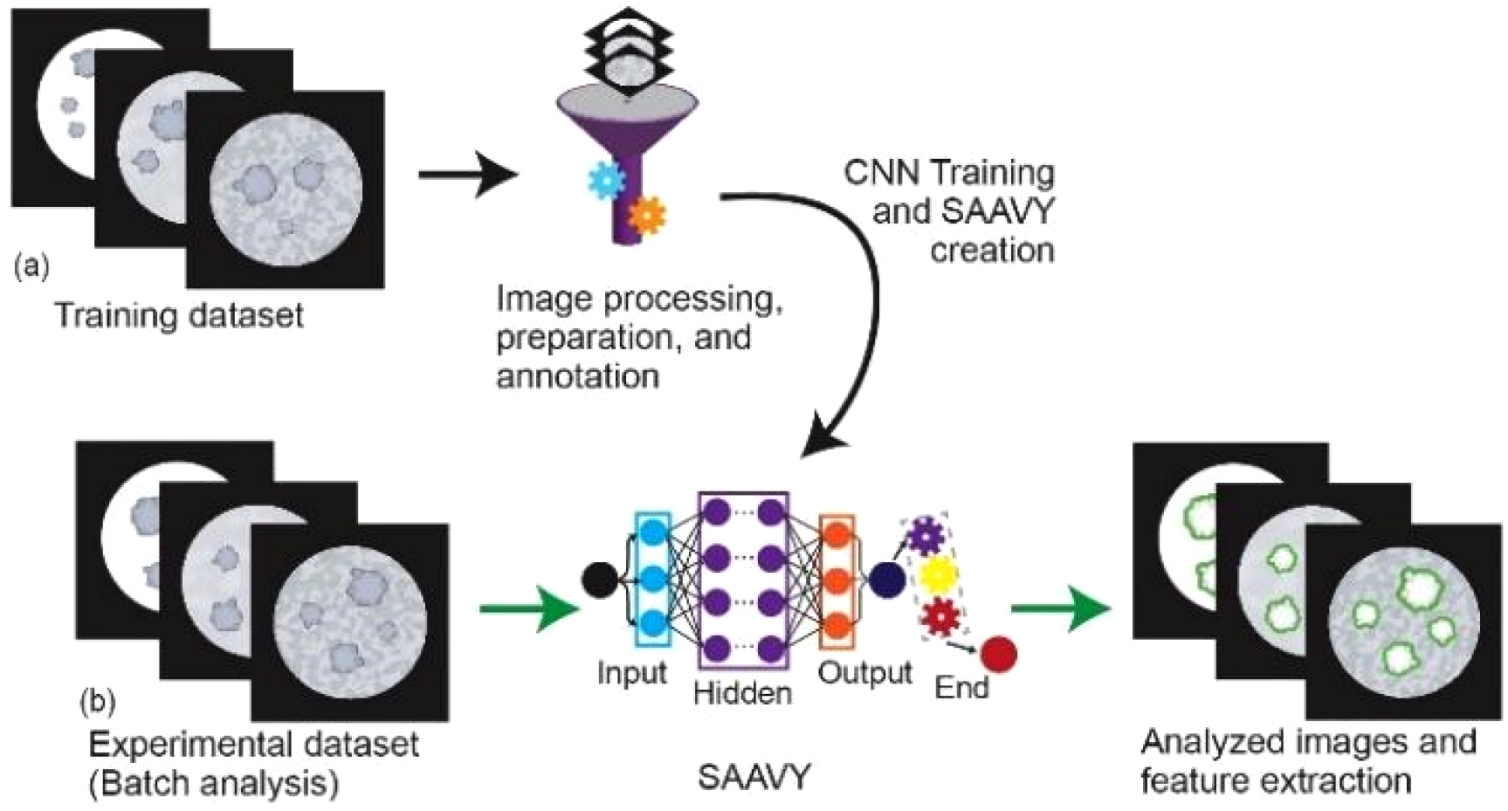
Overview of the SAAVY Training and Validation approach. (a) The training method for SAAVY is identified where we processed the images saved in the png file format for annotation and user-supervised training. The viability algorithm is developed during this step based on expert-determined characteristics for generating viability estimates. However, to avoid overfitting, the spheroid experts were not directly involved in the training. The training dataset was 30 images and required approximately 1 h. (b) Batch analysis was run on the experimental (validation) dataset images passed through SAAVY, which identified the mask of the region of interest in green and output a .csv file with other measured information about the image. Analysis time per image was 0.3 s. Both training time and analysis time are dependent on the computational power available and could be further accelerated.

An overview of SAAVY is presented in Fig. 1, and details are included in the supplementary material. SAAVY is first created by fine-tuning a pre-trained Mask R-CNN model in PyTorch.^58^ This implementation is particularly attractive due to its improved ability to separate overlapping features. This transfer learning approach reduced the total cost for bespoke images of cystic spheroids and allowed us to move forward with a relatively small number of expert-annotated images.^59^ We further refined our model with a balanced dataset consisting of 24 images of PDAC spheroids with clear and noisy backgrounds that spanned the entire viability range. The details on this process are contained in the supplementary material.

SAAVY analyzes brightfield, label-free images of tissue culture spheroids by segmenting each individual spheroid and outlining the identified region on the output image. Subsequently, SAAVY quantifies the viability of each uniquely identified spheroid using the average intensity of the segmented region compared to the background using a weighted average model as detailed in the supplementary material. This approach allows for an increased level of background noise to be present without negatively impacting the assessment ability. Several other metrics are calculated by SAAVY including the average viability, the average spheroid size, and total spheroid count across each image. With our hardware configuration, each image required 0.3 s for the entire analysis process. While viability assessments of 3D cultures are routinely performed as a quality check during experimentation by experts, the single-spheroid level quantitative analysis across an entire well is not easily obtained from expert analysis and is not possible using biochemical measurements. Therefore, SAAVY provides several orthogonal dimensions for analytics on an accelerated timescale.

The initial training and validation dataset contains a series of label-free microscopy images of PDAC spheroids cultured either in a clear or a noisy culture matrix. The images were annotated by the programming expert with no input from either spheroid expert. The noisy matrix was created by intercalating opaque nanoparticles throughout the gel. Images are acquired at days 0, 4, and 6 of spheroid growth at 4× magnification, and each image captures the entire 10 *μ*l gel seeded in a 96-well plate. The resultant dataset contains 1328 widefield images taken using an ECHO Revolve microscope. Notably, no images or wells were rejected from the dataset. Additional experimental details are included in the supplementary material.

**FIG. 2.**
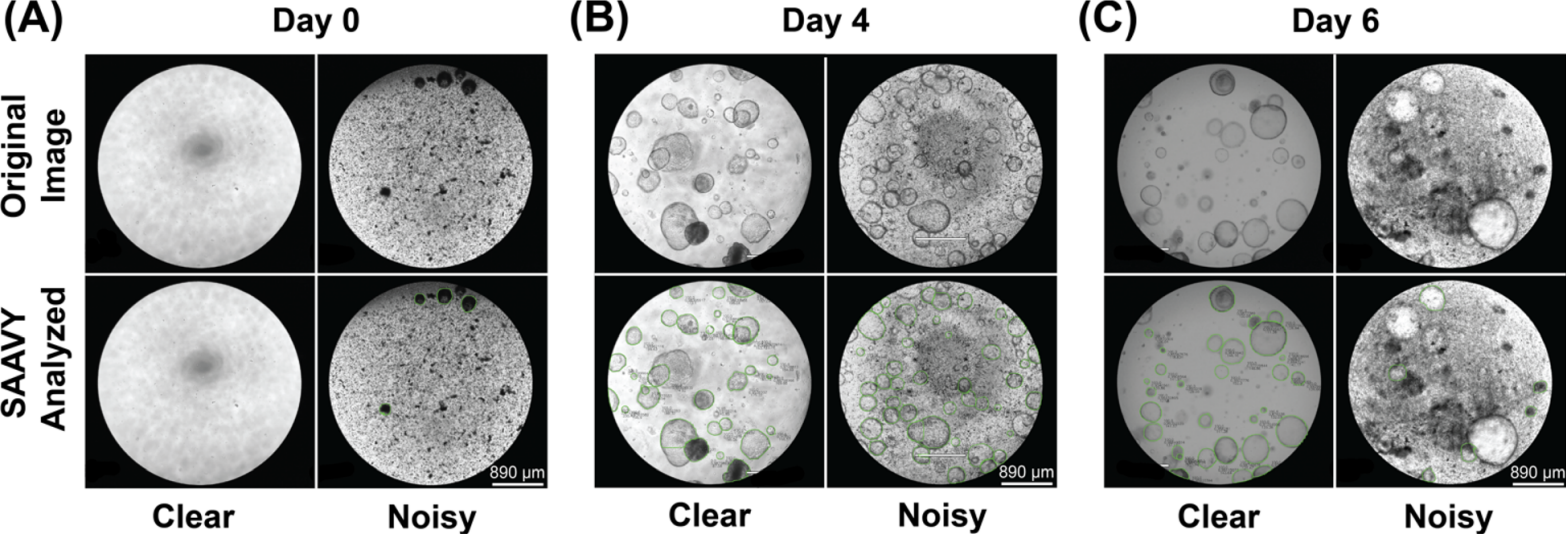
Images representative of before (top row) and after (bottom row) SAAVY analysis. The images are further subset by day: (a) D0, (b) D4, and (c) D6. For each day, both clear (no added nanoparticles) and noisy (5 mg/ml of added nanoparticles) images are shown. These are representative images selected from the total 416 images analyzed. Scale bar is 890 *μ*m for all images.

Representative examples of SAAVY-analyzed images across all days and both clear and noisy backgrounds are presented in Fig. 2. Both the original and the analyzed images are shown. As can be seen, SAAVY is able to identify spheroids in the presence of potential confounds and in cases where spheroids are overlapping. Notably, the entire image was analyzed at once. As a part of this work, SAAVY detected and provided information about 114 726 unique spheroids across the 2792 images taken on either an ECHO Revolve or an Operetta CLS microscope. The compatibility with two different imaging systems demonstrates its potential impact on the field.

The overall predictive accuracy of SAAVY was determined by calculating the intersection over union (IoU) for a subset of images randomly selected from the dataset highlighted in Fig. 2 for the ECHO Revolve and from a different dataset for the Operetta. The average IoU for the ECHO is 0.622, and the average IoU for the Operetta is 0.653. The details on this method and additional analysis are included in the supplementary material.

### B. SAAVY to expert comparison

SAAVY performance was evaluated as compared to a pair of blinded spheroid experts for the entire image population of clear and noisy background gels. The justification for expert selection and the blinding method is detailed in the supplementary material. Additionally, before comparing the expert assessments, their findings were harmonized. The steps involved in this process are detailed in the supplementary material.

Two indicators of SAAVY performance are evaluated: spheroid/no spheroid identification (ID) and live/dead detection (LD). ID is characterized as SAAVY assigning the appropriate value to each image according to the absence (value of −1) or presence (values of zero and above) of spheroids in the image. LD is characterized as SAAVY assigning viability values that correlate with live (>0%) or dead (0%) spheroids. This corresponds to the typical binary interpretation of other viability assays to keep analysis consistent with previous research. On days 4 and 6, ground truth was determined by expert 1, and the details for this decision are included in the supplementary material. Both ID and LD performance are quantified using the F1-score and summarized in Table I.

**TABLE I.** Summary of calculated metrics comparing spheroid expert 1, spheroid expert 2, and SAAVY throughout each sample sub-grouping (day and background type). Spheroid identification (ID) and live/dead (LD) analysis across all days and background types are presented first. Ellipses (...) are noted in columns where that method was used as either the ground truth (expert 1) or standard for comparison (SAAVY). Similarity and reliability are then quantified for each distribution comparison. We used the Earth Mover's distance to quantify the similarity between the two distributions and Krippendorff's alpha to quantify the reliability of each expert. Equations for accuracy and F1-score calculations are included in the supplementary material. Red cell highlights indicate better performance, whereas blue values indicate weak performance.

	Day 4					
	Clear			Noisy		
	Expert 1	Expert 2	SAAVY	Expert 1	Expert 2	SAAVY
ID F1-score	⋯	1	1	⋯	0.992	0.992
LD F1-score	⋯	1	1	⋯	0.992	0.992
Similarity	0.029	0.073	⋯	0.021	0.036	⋯
Reliability	0.531	−0.202	⋯	0.869	0.735	⋯
	Day 6					
ID F1-score	⋯	1	1	⋯	0.984	0.992
LD F1-score	⋯	0.914	0.891	⋯	0.962	0.954
Similarity	0.045	0.048	⋯	0.016	0.011	⋯
Reliability	0.869	0.861	⋯	0.863	0.854	⋯

The quantification of differences between the distributions was first calculated to compare SAAVY to each expert. For this quantification, Earth Mover's Distance (EMD) was used.^60^ Values of 0 correspond to less distance between the distributions, or classically that the effort is minimized to transform one distribution into the other. The EMD values are represented by the ‘similarity’ row in Table I. Further, the reliability was quantified using Krippendorff's alpha, a measure of inter-rater reliability. Values close to 1 suggest perfect reliability, whereas values closer to zero and negative values suggest poor reliability and systemic differences, respectively. The supplementary material contains a comparison of other common metrics and data correlations that we calculated for this analysis.

On day 4, SAAVY shows a decrease in reliability compared to expert 2 in clear samples (Table I). The same is not true for SAAVY as compared to expert 1 in clear backgrounds, and the reliability is improved for both experts for noisy background gels. The similarity also follows this trend for day 4. On day 6, SAAVY is nearly equally reliable when compared to both experts for both background types. However, the SAAVY distribution is more like the spheroid experts for noisy backgrounds as compared to clear backgrounds. The distributions are visualized for all permutations in the supplementary material for further comparison.

Day 4 showed clear improvement for both experts and SAAVY with F1-scores of 1.0 for both ID and LD analyses, which suggests perfect agreement with the ground truth. When spheroids are observed after the given time for growth, they are distinguishable from the background. The fact that SAAVY matches experts at this task suggests that the algorithm can successfully identify and analyze spheroids in clear backgrounds comparably well to human experts. LD analysis matched this trend of perfect F1-scores (1.0) for both SAAVY and expert on day 4. For noisy backgrounds, the same holds true though the F1-score decreases slightly to 0.992. Day 6 is when differences between SAAVY performance and the experts begin to appear. Notably, SAAVY outperforms expert 2 as compared to ground truth for spheroid identification. For LD analysis on clear backgrounds, it is 0.91 for expert 2 compared to 0.89 for SAAVY. On noisy backgrounds, this is further improved to 0.96 for expert 2 and 0.95 for SAAVY.

Although SAAVY has slightly lower F1 scores than expert 2 for live/dead performance, this difference is likely an artifact related to the size of detectable viability differences characteristic to each expert. SAAVY assigned viability values to a one-tenth of a percent as a direct scaling of the viability per pixel is 0.4%. Additionally, as can be seen in Fig. S5, SAAVY did not exhibit a bias in assignments toward any viability range. In contrast, after data harmonization, the spheroid experts classified viability in 10% increments. As a result, any spheroid with a viability below 10% is classified as dead. Additionally, the distribution of viability scores was biased toward the extreme ends. Therefore, SAAVY may classify an image of spheroids as alive although they are noted dead by experts due to its improved incrementation and non-biased assessment. To test this hypothesis, a ROC threshold analysis was completed to evaluate the level at which the threshold for live or dead can be raised to while optimizing the F1-score for SAAVY in clear samples. The threshold for SAAVY can be raised to 2.43% for clear backgrounds leading to an F1-score of 0.91, which matches expert viability prediction while maintaining improved resolution.

### C. Comparison against industry standard viability assay

CellTiter-Glo (CTG) is a metabolic viability assay that has been adapted and optimized for use when analyzing 3D tissue culture systems.^39,40^ Unlike image analysis methods, CTG relies on correlating the amount of cellular ATP released from lysed cells to the intensity of the luminescent signal generated by an enzymatic reaction. These assays are evaluated on a well-by-well basis through normalization of the well signal to positive and background control measurements. Although this assay is accepted and widely used as the industry-standard, previous research utilizing this method has highlighted several limitations. For example, specific metabolic effects may be obscured when measuring viability on its own.^61–63^ Inconsistent experimental procedures are also of concern for CTG measurements. Previous studies suggest measurements are dependent on variables such as the shaking time and wait time for equilibrium before measuring the plate luminescence.^39^ This variation may lead to errors in the normalization process standard for reporting the results of this assay. Image-based methods remove the question of experimental variation on reported viability.

We compare SAAVY viability to CTG viability measurements through a non-inferiority study to test if SAAVY performs no worse than the CTG method at characterizing viability. PDAC samples grown in either clear or noisy matrices were analyzed by the experts, CTG, and SAAVY. This approach resulted in 136 images with matched CTG data. Without noise in the matrix, the CTG viability reading exceeded 100% a total of seventeen times (24.3% of the measurements). Once matrix impurities were introduced, the CTG viability reading exceeded 100% in 39 samples (57.4% of the measurements). It should be noted that prior research has shown an increase in metabolic activity when grown in similar nanoparticle matrices,^64^ so the CTG results agree with prior findings.

**FIG. 3.**
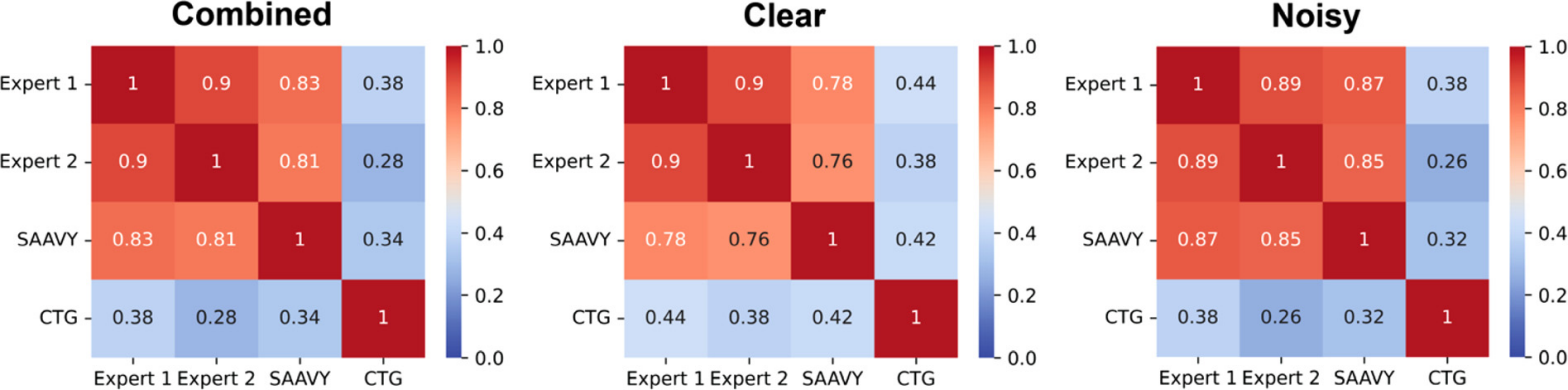
Pearson's R correlation analysis presented as heatmaps comparing experts, SAAVY, and CTG for the overall (combined), clear background, and noisy background datasets. Red indicates R values closer to 1 (perfect, positive correlation), and blue indicates no correlation. CTG values are poorly correlated with all other measurements regardless of the data subset. This is likely due to the relative interpretability of CTG results.

We compared CTG to SAAVY using Pearson's correlation, Earth Mover's distance for similarity, and Krippendorff's alpha for rater reliability across day 6 data for clear and noisy backgrounds. Figure 3 summarizes the results of the correlation comparisons. SAAVY and CTG have similar distributions for clear and noisy background types with EMD values of 0.023 and 0.010, respectively. We believe this agreement is due to the absence of spheroids in some images in this dataset, as the noisy background either obscured detection or killed the growing spheroids by this day. Figure S16 includes a histogram breakdown of these data to visualize the distributions. The reliability analysis, however, suggests that CTG is a more reliable rater on clear gels (k-alpha, 0.384) compared to noisy (k-alpha 0.076).

We could not complete a confusion matrix analysis for SAAVY and CTG because CTG cannot produce a negative result. We perform a threshold analysis on CTG to determine what the viability percent could be raised to with the goal of enhancing the overall performance of the CTG test. However, because of the inability of CTG to provide negative (0% viable by LD analysis) measurements, we looked to a metric other than F1-score during our threshold analysis. Youden's J-statistic was used to determine that the cutoff for CTG can be raised to 18.00% and 48.15% for clear and noisy backgrounds, respectively. This suggests that the CTG assay is not specific when capturing cell state characteristics and points toward the potential impact of an image-based companion for viability measurement.

### D. Nondestructive tracking of perturbation longitudinal impact on 3D cultures

One current hurdle in longitudinal studies using high-throughput, automated 3D culture methods is the requirement of labeling the sample to assess viability. A nondestructive, label-free approach would allow continuous monitoring of the same spheroid, increasing research rigor and allow primary cells and samples to be used. As a step in this direction, SAAVY's ability to longitudinally track PDAC spheroid growth within the same sample is demonstrated by analyzing label-free images and creating a viability response curve.

**FIG. 4.**
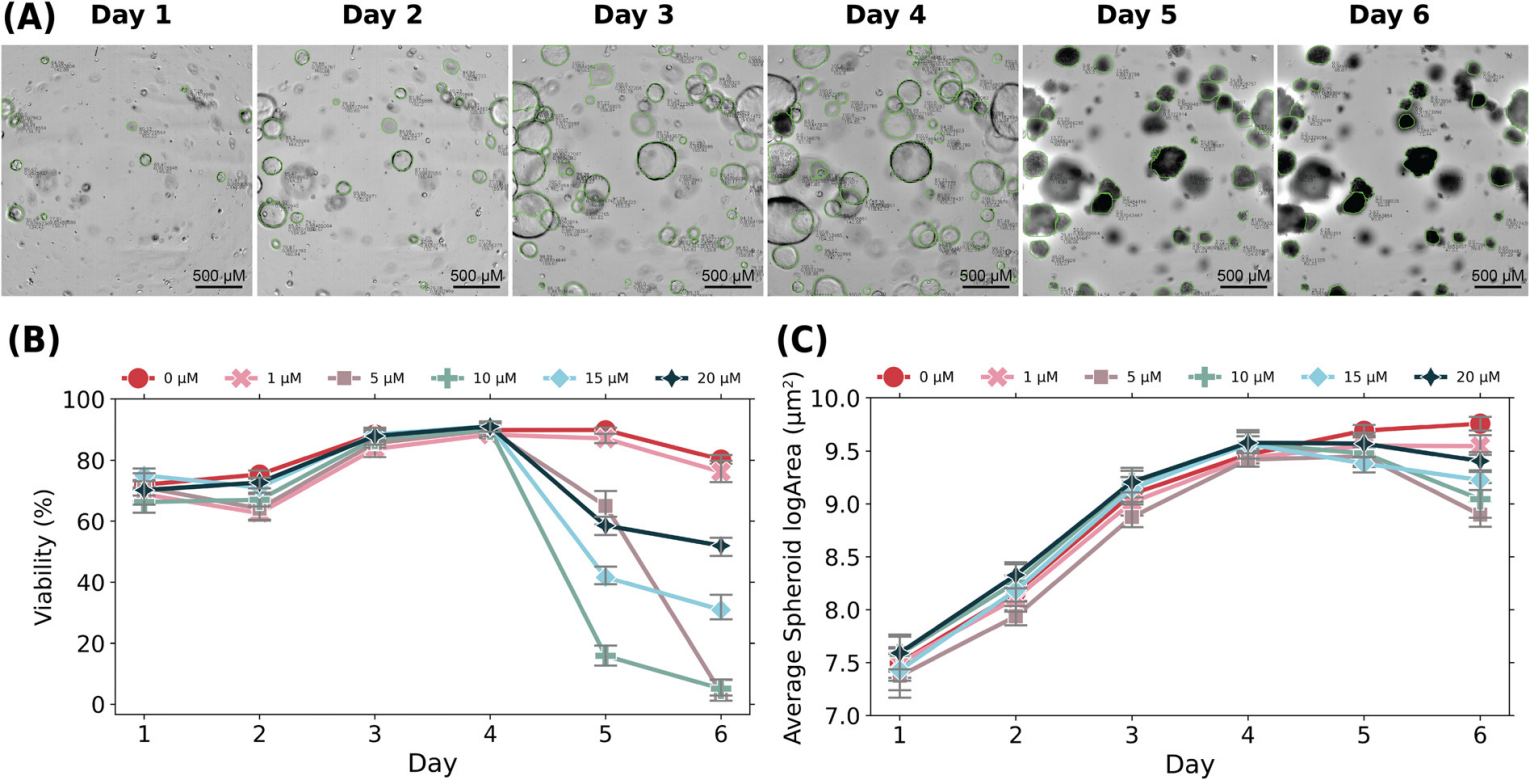
Longitudinal analysis of PDAC response to gemcitabine evaluated at whole-well resolution. (a) Images on the top row depict a representative sample well during each day of a six-day dose-response assay. The chosen well was perturbed with 10 *μ*M drug (gemcitabine) on day 4 after imaging. Images are of the same focal plane (320 *μ*m) throughout the course of the experiment. The scale bar is 500 *μ*m. (b) and (c) Line plots of SAAVY application to analyze the viability and growth of spheroids at whole-well resolution across the dose-response experiment where: (b) the raw viability of each drug-response group over the assay and (c) the average log-transformed spheroid area (*μ*m^2^) of each drug-response group over time.

An Operetta CLS high content imager is used to automate the image acquisition process. During this experiment, we took z-stack images of each well in a 96-well plate 20 *μ*m apart from 0 *μ*m at the hydrogel/well-plate interface to 700 *μ*m at the top of the hydrogel resulting in 36 images per each well. Imaging for this experiment was conducted on six days following the seeding of the experimental plate on day 0. The final longitudinal image set included 12 528 images where 2376 images were selected for analysis (105 723 spheroids). The z-height used in Figs. 4(a) and 4(c) was located at our chosen mid-plane in the well at 320 *μ*m. Five planes above and below the midplane were included for individual spheroid analyses. However, to scan the largest region within the center of the gel to test uniformity of SAAVY analyses, we chose planes 40 *μ*m apart. Further details are included in the supplementary material.

Gemcitabine, an anti-metabolite therapeutic commonly used to treat pancreatic cancer, was used for compound perturbation at concentrations of 1, 5, 10, 15, and 20 *μ*M. We included wells of untreated PDAC spheroids for a positive control. Treatment was added after imaging on day 4 to all wells using appropriate volumes of a stock 100 *μ*M solution of gemcitabine in DMSO. An example well from the 10 *μ*M perturbation group was highlighted in Fig. 4(a) to note the change in morphology and color of spheroids over time in this experiment.

Whole-well viability, plotted by treatment group, is presented in Fig. 4(b). Critically, we did not normalize the reported viabilities in this plot and used the raw output from SAAVY to emphasize the low error within repeated sample conditions. The decreased viabilities reported by SAAVY on days 1 and 2 is likely due to the smaller size of the spheroids at this time, specifically taking into consideration that our training was done on day 4 and day 6 spheroids. However, the stability of viability across days 3 and 4 of spheroid growth is suggestive of the overall reliability of SAAVY to track samples across different days.

SAAVY detects viability responses across all sample groups. The untreated and 1 *μ*M groups trend similarly for viability across all days. Interestingly, the mechanisms of compound diffusion are potentially identified in our image-based assay as well. We note a delayed spheroid-death response from the 5 *μ*M concentration, perhaps due to diffusion time through the gel leading to decreased availability of compound 24-h after treatment compared to 48-h post-treat. In contrast, we see a smaller change in viability across the 15 and 20 *μ*M concentrations. Upon qualitative analysis of the images, we observed a distinctly different morphology of the spheroids. Spheroids at these higher compound concentrations look like they have collapsed on themselves, compared to the lower (5 and 10 *μ*M) where the spheroid looks like it has exploded and has characteristic blebbed edges suggesting cell apoptosis [as shown in the day 5 and day 6 images in Fig. 4(a)].

We investigate if the decreased viability trends with spheroid area, as we expect the area to decrease as the spheroids die. This is confirmed by visualizing the average logarithmic transformed area across each day of the assay, seen in Fig. 4(c). We see an upward trend in spheroid growth across all treatment groups through day 4 of the assay. This metric allows us to investigate further trends in spheroid response to compound treatments. For the control group, we see continued growth through the final day of imaging. We see an expected decrease in the spheroid size from day 4 to day 6 in the 5, 10, 15, and 20 *μ*M treatment groups that correlates with the decreased viability seen on these days. For the lowest concentrations of treatment, the size increases until day 5, though at a slightly lesser rate than the control group, where it then stays constant between day 5 and day 6. When taken into context with the viability data, this suggests that this smaller concentration of compound may be enough to stall continued growth without eradicating the tumor cells. A potential rate-based result underscores the important role that image-based surveillance methods play during cell viability experiments.

**FIG. 5.**
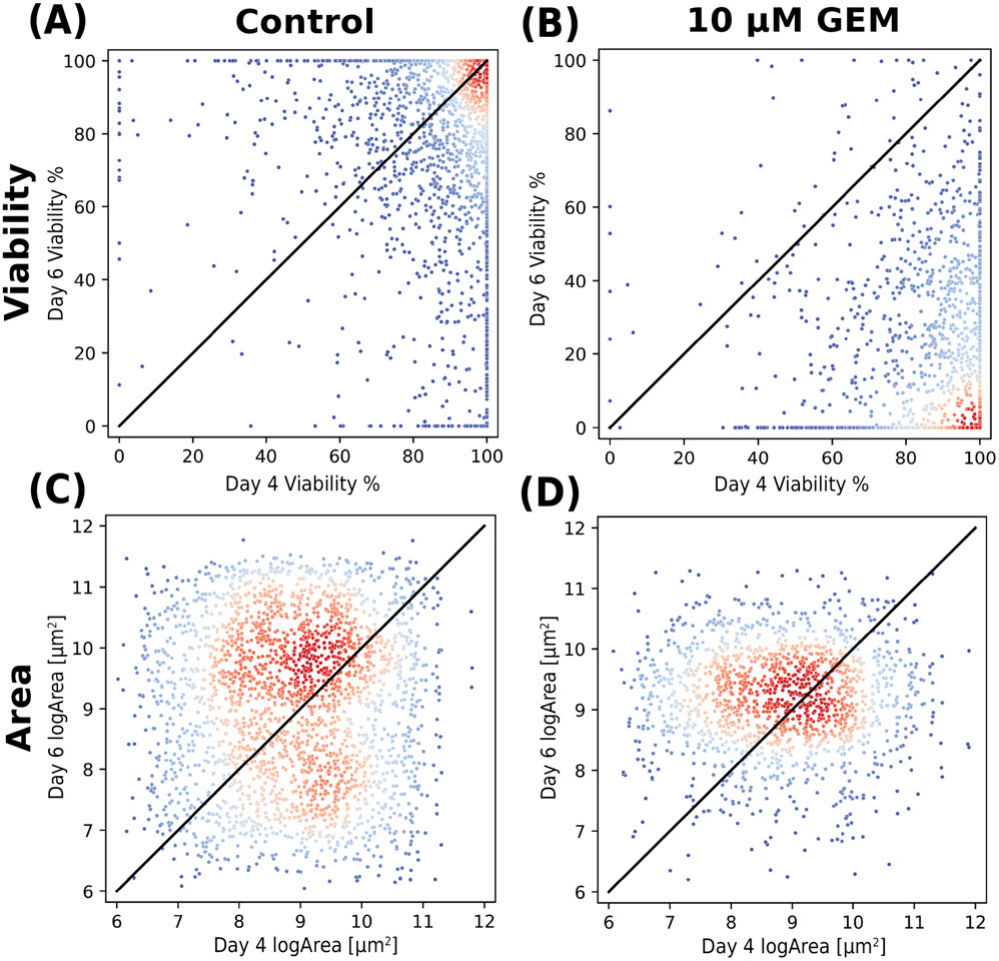
Scatter plots of individual spheroids from day 6 plotted against day 4 spheroids colored according to a Gaussian kernel density estimation where blue is lower density and red is higher density. The plots either present the viability (a) and (b) or the log(Area) (c) and (d) for either the control group or the 10 *μ*M gemcitabine (GEM) concentration perturbation group.

To further investigate these rate-based changes, we performed a single spheroid level analysis, and we plot day 6 metrics against day 4 metrics on spheroids taken from the same sample. The scatterplot representation in Fig. 5 underscores the visual changes that take place in the sample with the introduction of perturbation compound. The black diagonal line in each plot is a guide to the eye. Any spheroid above the line is increasing in viability or area from day 4 to day 6; conversely, data below the line have experienced a decrease.

In the viability plots [Figs. 5(a) and 5(b)], we see the highest density of spheroids, represented by the red colored points on the scatter, moving from the top right of the plot in the control group to the bottom right of the plot in the 10 *μ*M concentration group. This distribution shift clearly highlights the negative viability trend in individual spheroid response. The area plots in Figs. 5(c) and 5(d) show an interesting nuance to the spheroid size. Where the whole-well analysis suggests that the area of spheroids decreases with perturbations and cell death, additional information is revealed when the data are analyzed at the single spheroid level.

Specifically, in the control group, two spheroid size populations are clearly identifiable. The larger of the two is increasing in size, but a small population is decreasing. In contrast, in the GEM-treated samples, there is a uniform size population that is stagnant in size. When analyzed in conjunction with the data in Fig. 5(b), one possible conclusion is that these spheroids are all dead. This type of analysis that blends population-level and single spheroid level data is not possible with other approaches and opens the door to reveal new mechanistic insights.

**FIG. 6.**
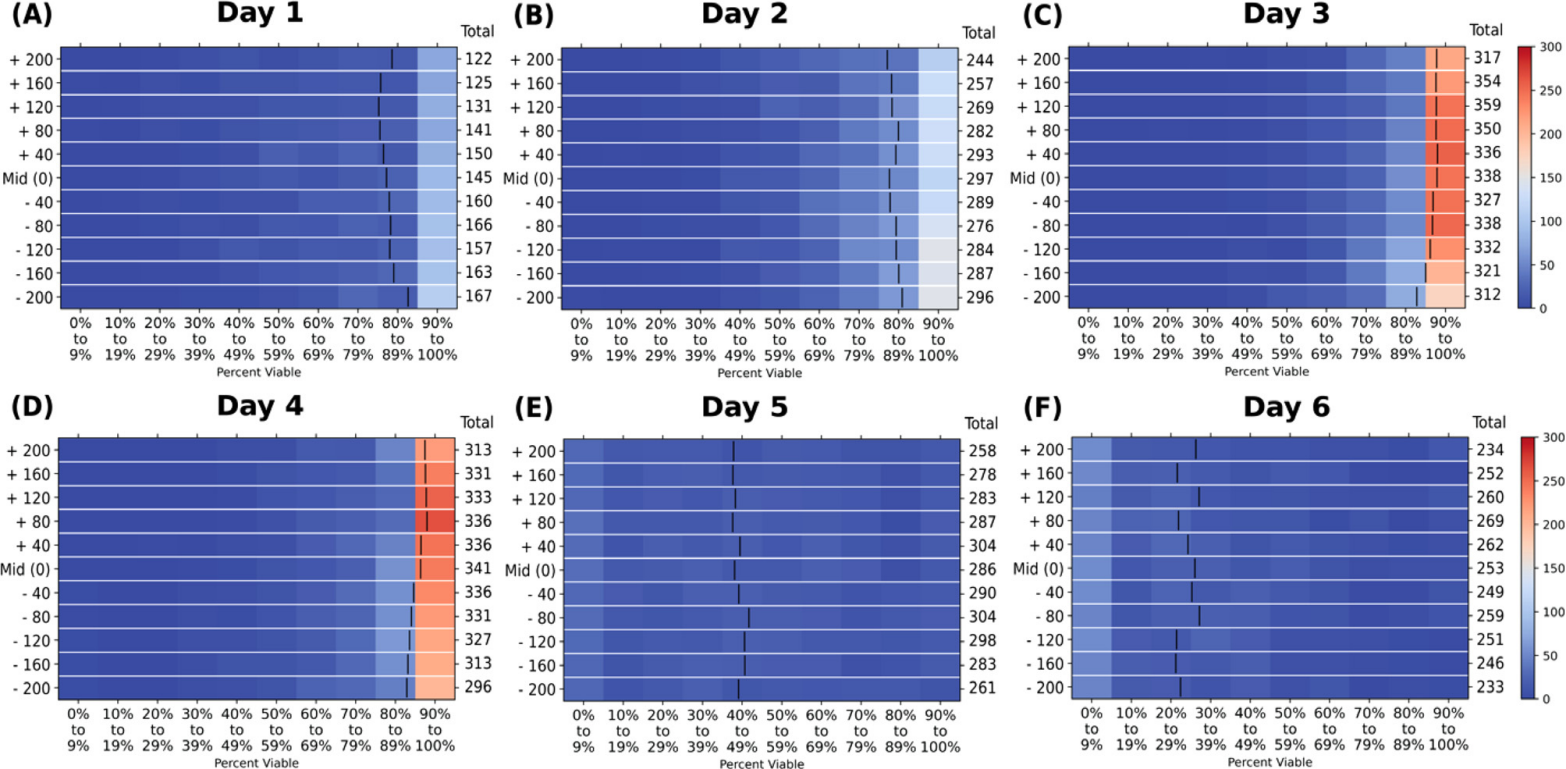
2D histograms of spheroids in the 10 *μ*M perturbation group separated by the focal plane from imaging against the percent viability (binned in 10% increments) of all individually identified spheroids in the dataset. The total number of spheroids identified per each plane is noted in the “total” column between the plot and the color bar. The black bar within each plane grouping is the group's average viability. The color bar represents the total number of spheroids within each viability bin, where red colors indicate high counts (closer to 300) and blue indicate low counts (closer to 0) of spheroids within each of the viability bins. Data are segmented by day accordingly. Subplots are for the day of experiment: (a) day 1, (b) day 2, (c) day 3, (d) day 4, (e) day 5, and (f) day 6.

To round out the analysis, we compare images at different z-planes within the well and present these data as 2D histograms separated by day in Fig. 6. We analyzed five image planes above and below the mid-plane, which was the data presented and analyzed in Fig. 4. Each subsequent plane was 40 *μ*m separated from the one before it to span the middle region of the spheroid-laden hydrogel. This spacing avoids interface effects, allowing us to capture the bulk characteristics. Further details are included in the Supplemental Information.

We extracted information on each individual spheroid within the images corresponding to the plane. All the 105 723 segmented spheroids are present in the plots detailing this analysis, with the other perturbation groups and area visualizations included in the supplementary material. In Fig. 6, we see the same trend of increasing spheroid viability across days 1–4 until perturbation, followed by decreasing viability on days 5 and 6. Importantly, we note the consistency of SAAVY to assign variabilities within each of these planes. The average for each plane distribution is consistent across each grouping and each day.

The individual spheroid analysis presented in Figs. 5 and 6 suggests that SAAVY can uniformly identify the overall spheroid population within the growth matrix, and SAAVY analysis does not lose sight of overall response dynamics of spheroids within the entire culture.

## III. CONCLUSIONS

We develop and validate a method to quantitate viability of 3D tissue cultures in a label-free, nondestructive, and longitudinal manner that easily integrates into existing tissue culture imaging procedures. We showed comparable quantification using our algorithm, SAAVY, to human expert estimation in both clear and noisy background hydrogels with high tolerance to noise. We found improvements in classification ability and sensitivity over standard CTG assay measurement. Further, we showed the ability of SAAVY to longitudinally track 3D spheroid growth and viability for both untreated and therapeutically treated spheroids. Our algorithm uses the same principle that human experts use to evaluate images yet streamlines the process by automation. This facilitates integration of our viability method into standard tissue culture procedures in a robust and time-independent manner. The ability of our algorithm to output results of “no spheroid” as well as quantitative morphological metrics when analyzing 3D tissue culture constructs adds another analysis level on top of typical live/dead classification and can provide more information to an experimentalist regarding growth characteristics of a sample.

With the augmentation of biomedical imaging with computer vision and other CNN approaches, it is possible to further develop label-free imaging methods. Our system only covered the supervised training of one tissue spheroid type, PDAC. The open-source nature of our algorithm may allow for user-specified training of different kinds of 3D spheroid culture images as well as tunability of the viability algorithm to suit the characteristics of other cultures. There is opportunity to expand the training data of SAAVY beyond one-spheroid type and utilize other deep-learning methods, such as unsupervised learning, to expand the capabilities of SAAVY. This work considered the plane of best representation of each imaged spheroid in the experimental plates. To truly evaluate efficacy of compounds and materials, it is important to utilize the full reconstructive capabilities when merging 3D imaging with deep learning. The area analysis in this study and other research points toward the importance of the ability to study fully reconstructed 3D cultures in supporting drug discovery and uncovering drug affects on overall growth dynamics.^65^ In this context, improved monitoring may improve rigor in its ability to compare across experiments. The time-agnostic and nondestructive manner of this algorithm quantifies the qualities that researchers often judge when monitoring their culture. With further expansion of our training dataset to include various disease types and 3D tissue culture systems, we believe that SAAVY may prove a useful tool for real-time analysis and can complement image-based 3D culture assays where viability must be assured.

## IV. METHODS

### A. Tissue culture

Mouse-derived pancreatic cancer (PDAC) spheroids (line 8-14F-7: KRAS G12D, PTEN loss, COX2 overexpression, female, 2 weeks old at the time of sacrifice) were used. Pancreatic cancer spheroids were cultured using an established protocol^66^ included in the supplementary material. For samples that do not include any nanoparticles, the cell-laden hydrogel solution was plated in 10 *μ*l increments in the center of the wells on an opaque-walled 96-well plate (Corning). For samples that included nanoparticles, nanoparticle solutions were combined with cell-laden solutions to the desired concentration of cells and nanoparticles then plated in the same manner mentioned above.

Images were taken of all seeded wells on the day of seeding (day 0, D0). The plates were left to incubate until day 4 (D4) where negative control wells were treated with 10 *μ*M gemcitabine added to the cell media and images were captured of all wells. We incubated the plates for an additional two days until day 6 (D6) where we took final images of all then performed a 3D CellTiter Glo (CTG) assay (Promega) to measure the metabolic activity of the spheroids grown in each condition. CTG was performed according to the documented protocol provided by Promega and read using a BioTex plate reader for luminescent detection at 560 nm. Dose–response assays were plated in the same manner described above and imaged at 320 *μ*m above the bottom of the well plate for consistent cross sections across all six days of experimental growth. Images were exported from the Operetta CLS microscope with a brightfield correction factor applied to images to reduce vignetting from background light within the imaged gel.

### B. Image datasets

Our datasets include brightfield images taken during various experiments, as noted in the tissue culture methods section above. PDAC images were taken on an ECHO Revolve, 4×/0.13 objective lens, or Operetta CLS with 5×/0.16 objective lens. ECHO images were saved and exported in TIFF format. Images from CLS were exported as PNG with a brightfield correction applied by the instrument software to remove a vignette from the instrument's inhomogeneous light source. Both were converted to JPG and digitally resized to 1024 × 1024 for SAAVY viability analysis. The complete PDAC set included 1328 images. Of these, 24 images were randomly selected for training, and 416 images were used for the experimental viability estimations where 136 were CTG matched. The longitudinal dataset included 12 528 images where 2376 were selected for analysis.

### C. Data training, pre-processing, and final run time

We first train SAAVY using a pre-existing image set from MS COCO pretrained general model for transfer learning. We trained for 20 epochs. Based on analysis, we used the 15th checkpoint for image production due to continuous loss after this point (Fig. S3). Using the training dataset detailed above, we annotate the 24 training images using VIA Image Annotator (2.0.11, Oxford) to specifically identify cell spheroids. The total computational time for SAAVY on the 416-image set is 2 min and 5 s (running an RTX 3080 and Intel Core i9-10850K stock).

### D. Post hoc analysis

We assess confusion matrices for two classifications: spheroid/no spheroid and live/dead detection. Accuracy and F1-score (SI, EQ-3,4) were calculated where appropriate to get an overview of the performance of SAAVY as compared to experts and how the experts compare to each other. We assessed data matching by first converting data to their respective probability density function and then calculating the multiple distance metrics to assess the distances between all viability estimation methods (Table S5).

Before performing any statistical analyses, we assessed overall normality of the data according to the group analyzed. Where appropriate, non-normal data were estimated normal according to the central limit theorem and the appropriate groupwise analyses were applied (either repeated measured analysis of variance with Tukey post hoc test or related t-test for normal data or Wilcoxon for nonparametric data from small samples). All analysis was completed in Python with the appropriate libraries noted in the supplementary material.

## SUPPLEMENTARY MATERIAL

See the supplementary material for the following: (1) 3D culture and experimental preparation methods, (2) dataset generation, (3) SAAVY development, (4) analysis, and (5) experimental metadata, and also extensive data from our single spheroid and planar analysis in additional plots.

## Data Availability

The data that support the findings of this study are openly available in Zenodo at http://doi.org/10.5281/zenodo.10086367, Ref. 67, and GitHub at https://github.com/armanilab/SAAVY, Ref. 68.
